# iEnhancer-ECNN: identifying enhancers and their strength using ensembles of convolutional neural networks

**DOI:** 10.1186/s12864-019-6336-3

**Published:** 2019-12-24

**Authors:** Quang H. Nguyen, Thanh-Hoang Nguyen-Vo, Nguyen Quoc Khanh Le, Trang T.T. Do, Susanto Rahardja, Binh P. Nguyen

**Affiliations:** 1 School of Information and Communication Technology, Hanoi University of Science and Technology, 1 Dai Co Viet, Hanoi 100000, Vietnam; 2 School of Mathematics and Statistics, Victoria University of Wellington, Gate 7, Kelburn Parade, Wellington, 6142 New Zealand; 3 Professional Master Program in Artificial Intelligence in Medicine, Taipei Medical University, Keelung Road, Da’an Distric, Taipei City, 106 Taiwan (R.O.C.); 4 Institute of Research and Development, Duy Tan University, Danang 550000, Vietnam; 5 School of Marine Science and Technology, Northwestern Polytechnical University, 127 West Youyi Road, Xi’an 710072, China

**Keywords:** Enhancer, Identification, Classification, Ensemble, One-hot encoding, Convolutional neural network, Deep learning

## Abstract

**Background:**

Enhancers are non-coding DNA fragments which are crucial in gene regulation (e.g. transcription and translation). Having high locational variation and free scattering in 98% of non-encoding genomes, enhancer identification is, therefore, more complicated than other genetic factors. To address this biological issue, several in silico studies have been done to identify and classify enhancer sequences among a myriad of DNA sequences using computational advances. Although recent studies have come up with improved performance, shortfalls in these learning models still remain. To overcome limitations of existing learning models, we introduce iEnhancer-ECNN, an efficient prediction framework using one-hot encoding and *k*-mers for data transformation and ensembles of convolutional neural networks for model construction, to identify enhancers and classify their strength. The benchmark dataset from Liu et al.’s study was used to develop and evaluate the ensemble models. A comparative analysis between iEnhancer-ECNN and existing state-of-the-art methods was done to fairly assess the model performance.

**Results:**

Our experimental results demonstrates that iEnhancer-ECNN has better performance compared to other state-of-the-art methods using the same dataset. The accuracy of the ensemble model for enhancer identification (layer 1) and enhancer classification (layer 2) are 0.769 and 0.678, respectively. Compared to other related studies, improvements in the Area Under the Receiver Operating Characteristic Curve (AUC), sensitivity, and Matthews’s correlation coefficient (MCC) of our models are remarkable, especially for the model of layer 2 with about 11.0%, 46.5%, and 65.0%, respectively.

**Conclusions:**

iEnhancer-ECNN outperforms other previously proposed methods with significant improvement in most of the evaluation metrics. Strong growths in the MCC of both layers are highly meaningful in assuring the stability of our models.

## Background

‘Omics’ science, including studies on genomics, transcriptomics, proteomics, and metabolomics, is a new research field combining background of molecular genetics and power of computer science to address biological problems. In transcriptomics, enhancers [1] refer to a group of non-coding DNA fragments holding responsibility for regulating gene expression in both transcription and translation. Unlike a promoter which is the transcriptional initializer of a particular gene [2] located at the upstream region of the gene, an enhancer can be found at a region of up to 20kb upstream/downstream with respect to the gene or even at other chromosomes not carrying that gene. Identification of new enhancers is therefore challenging due to their nature of locational variation. Besides, since enhancers are sequences not encoding for any proteins, they freely dispense into 98% of the total human non-encoding genome carrying billions of base pairs [1]. While molecular mechanisms of protein-coding genes can be relatively simply addressed, biological patterns of enhancers have not been well generalized. Furthermore, activities of enhancers vary depending on specific types of cells, time, and intrinsic/extrinsic stimulations [1]. Previously, to identify and locate enhancers, scientists had no choice but performing in vitro [3] or in vivo [4] experiments. Recent findings have revealed there are a large number of recognized enhancers shared by both human and other species including eukaryotes and prokaryotes [1, 5]. Moreover, genetic variation in enhancers has been demonstrated linking to many human illnesses [6, 7] such as various types of cancer [6, 8] and inflammatory bowel disease [9].

As an essential transcriptional factor facilitating gene expression, enhancer identification/classification is currently one of hot topics in biological research that are appealing to both experimental and computational biologists [10–12]. In 2007, a comparative analysis on genomics was done by Pennacchio et al. [10] to identify enhancers. Since the study used a small training dataset, the limited prediction accuracy was one of their big challenges at that time. In 2017, Zacher et al. proposed a novel unsupervised genome segmentation algorithm called GenoSTAN (Genomic STate ANnotation) [11] to improve the accuracy in enhancer/promoter identification by directly learning from sequencing data of chromatin states (no data transformation required). GenoSTAN used 127 cell types and tissues collected from the ENCODE [13, 14] and NIH Roadmap Epigenomics Program [15]. Although their study using chromatin state data to identify enhancers ended up with good results, the model sensitivity was still lower than that of other methods using transcription-based data because transcription-based predictive models using transient transcriptome profiling [16, 17] and nascent transcriptome profiling [18] could significantly boost up the model sensitivity. A year later, Lai et al. [12] conducted wet-lab experiments to identify the enhancers of red flour beetle (*Tribolium castaneum*) and evaluated their activity.

Unlike in the past, computational scientists are now equipped with high-performance computing resources and advanced techniques to deal with the outgrowth of biological data, especially ‘omic’ data. Troubleshooting biological problems using various in silico approaches is one of the best ways to take advantages of redundant and available ‘omic’ data. For enhancer identification and classification, some in silico studies have also been conducted using genetic regulatory elements such as transcriptional factors binding motif occurrences [19], chromatin signatures [20], and combined multiple datasets [21]. To improve model performance, computational scientists have applied various learning algorithms, e.g. the Random Forest (RF) [22], deep belief networks [23], deep-learning-based hybrid [24] and neural network [20] architectures. In 2016, iEnhancer-2L [25] by Liu et al. and EnhancerPred [26] by Jia and He were introduced as two effective methods using the same learning algorithm - Support Vector Machine (SVM). While iEnhancer-2L used pseudo k-tuple nucleotide composition (PseKNC) for sequence encoding scheme, EnhancerPred used bi-profile Bayes and pseudo-nucleotide composition. Both methods reported acceptable performances; however, their MCCs were relatively low. EnhancerPred performs slightly better than iEnhancer-2L with small improvement in MCC; however, its efficiency is still insufficient. In 2018, Liu et al. proposed iEnhancer-EL [27] which is an upgraded version of iEnhancer-2L. It has a very complicated structure with two ensemble models from 16 individual key classifiers, and the key classifiers were constructed from 171 SVM-based elementary classifiers with three different types of features: the PseKNC, subsequence profile, and *k*-mers. Although iEnhancer-EL is currently one of the best methods for identifying enhancers and their strength, it should be possible to develop better models using novel learning algorithms and encoding schemes.

In this study, we propose a more efficient prediction framework called iEnhancer-ECNN using a combination of one-hot encoding (OHE) and *k*-mers as a sequence encoding scheme and ensembles of convolutional neural networks (CNNs). In order to make a fair comparison with other previous studies, the same dataset used in Liu et al.’s studies [25, 27] and Jia and He’s study [26] was used in our model construction and evaluation.

## Results and discussions

### Sequence analysis

To perform comparative sequence analysis on biological patterns between enhancers and non-enhancers as well as those between strong enhancers and weak enhancers, Two Sample Logo [28] with independent *t*-test (*p*<0.05) was adopted to generate a logo to visualize the sequence. An initial concept of presenting consensus sequences to visualize shared biological patterns in a set of aligned sequences was first proposed by Schneider et al. [29] in 1990. Each sequence-logo map displays information about (i) the most prevalently found nucleotides scoring from the head of each certain location, (ii) the occurrence frequency of every nucleotide signified by the proportional height of the character, and (iii) the significance of every particular location relying on by the height of the entire stack of characters.

For both layers in this study, a significance testing for the variance of biological patterns between enhancers and non-enhancers as well as between strong enhancers and weak enhancers was conducted. For layers 1 and 2, the enhancer set and strong enhancer set are considered positive sets while the non-enhancer set and weak enhancer set are considered negative sets. The constructed map for each layer provides information about two groups of nucleotides observed in the positive set and the negative set (base for comparison) sequentially. A nucleotide which is commonly detected in a certain location of numerous samples from the positive set is named ‘enriched nucleotide’ whereas a nucleotide which is seldom detected in a certain location of numerous samples from the positive set is named ‘depleted nucleotide’. Independent *t*-test was done using the calculated occurrence frequencies of a nucleotide at certain locations to gain information on which nucleotide occurrence is accidental or directional.

**Fig. 1:**
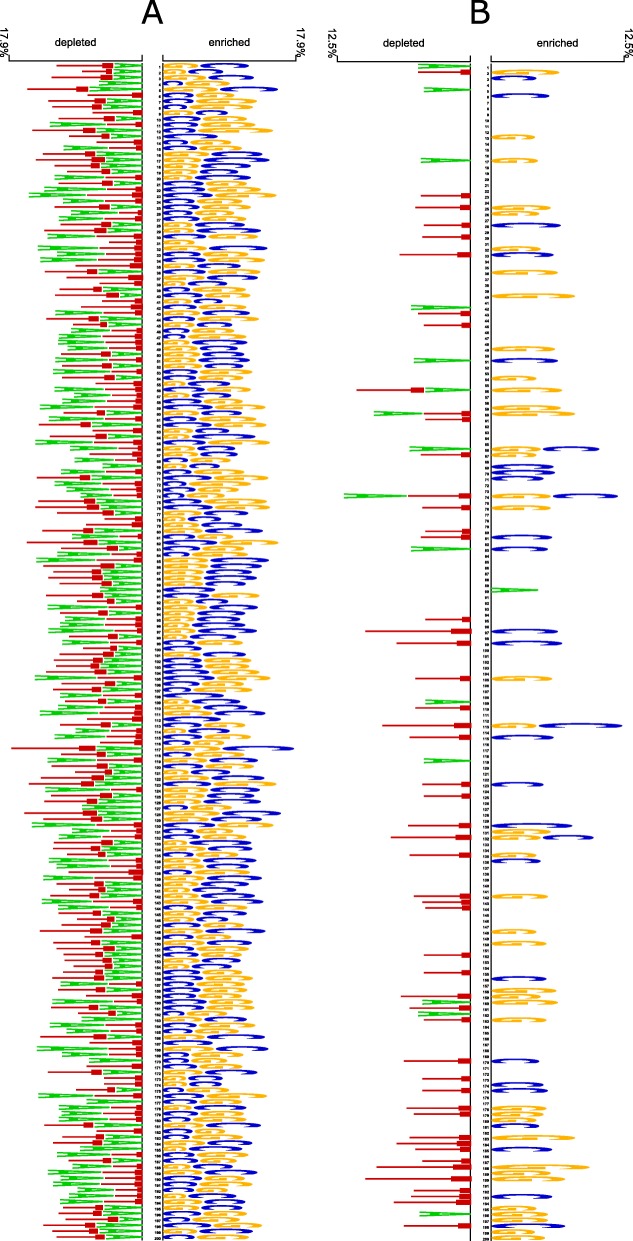
Sequence characteristics of **a** enhancers versus non-enhancers and **b** strong enhancers versus weak enhancers. Sequence analysis using logo representations were created by Two Sample Logo with *t*-test (*p*<0.05) with A, T, G, and C are colored with Green, Red, Yellow, and Blue, respectively

Figure 1 indicates sequence characteristics of sites between enhancers and non-enhancers and between strong enhancers and weak enhancers, respectively, in the development set. It is obviously seen that along most of the enhancer sequences, each location is enriched with only G and C while depleted with A and T. This significant difference between enhancers and non-enhancers indicates a great separation in biological patterns between two groups, or in other words, this finding is meaningful for our classification model. Besides, structural differences between strong enhancers and weak enhancers are evidently smaller than those between enhancers and non-enhancers due to many shared biological patterns. As shown in Fig. 1B, strong enhancers have a tendency to accumulate G and C more rather than A and T while weak enhancers show a completely reverse trend with a condensed population of A and T and a sparse population of G and C.

### Model evaluation

Tables 1 and 3 compare the performances on the independent test set of 5 single CNN models versus the ensemble model in layers 1 and 2, respectively, to examine the efficiency of using ensemble learning. Tables 2 and 4 provide information on 10 testing trials in layers 1 and 2, respectively. For each trial, a random seed in the range from 3 to 21 was used to split the development dataset into five parts using stratified sampling. Each part was in turn used as the validation set for training a CNN model from the remaining 4 parts.

**Table 1:** Results of an enhancer identification trial (trial 5 in Table 2) on the independent test dataset

Training : Validation (Ratio 4:1)	ACC (%)	AUC (%)	SN (%)	SP (%)	MCC
Model 1 (Parts 2, 3, 4, 5 : Part 1)	0.756	0.815	0.750	**0.765**	0.515
Model 2 (Parts 1, 3, 4, 5 : Part 2)	0.753	0.829	0.775	0.730	0.506
Model 3 (Parts 1, 2, 4, 5 : Part 3)	0.740	0.825	**0.810**	0.670	0.485
Model 4 (Parts 1, 2, 3, 5 : Part 4)	0.776	0.831	0.790	0.765	**0.555**
Model 5 (Parts 1, 2, 3, 4 : Part 5)	0.746	0.821	0.745	0.750	0.495
Ensemble Model	**0.765**	**0.834**	0.790	0.740	0.531

The highest value for each metric is in bold

**Table 2:** Independent test identifying enhancers and non-enhancers under 10 trials

No. of Trials	ACC (%)	AUC (%)	SN (%)	SP(%)	MCC
1	0.768	0.831	0.780	0.755	0.535
2	0.765	0.834	0.790	0.740	0.531
3	0.770	0.835	0.775	0.765	0.540
4	0.768	0.831	0.795	0.740	0.536
5	0.773	0.832	0.785	0.760	0.545
6	0.778	0.837	0.800	0.755	0.556
7	0.773	0.832	0.780	0.765	0.545
8	0.773	0.832	0.780	0.765	0.545
9	0.758	0.830	0.785	0.730	0.516
10	0.763	0.830	0.780	0.745	0.525
Mean	0.769	0.832	0.785	0.752	0.537
SD	0.006	0.002	0.008	0.013	0.011

**Table 3:** Results of an enhancer classification trial (trial 9 in Table 4) on the independent test dataset

Training : Validation (Ratio 4:1)	ACC (%)	AUC (%)	SN(%)	SP (%)	MCC
Model 1 (Parts 2, 3, 4, 5 : Part 1)	**0.700**	**0.764**	0.780	0.620	0.405
Model 2 (Parts 1, 3, 4, 5 : Part 2)	0.660	0.740	0.720	0.600	0.322
Model 3 (Parts 1, 2, 4, 5 : Part 3)	0.670	0.730	**0.850**	0.490	0.364
Model 4 (Parts 1, 2, 3, 5 : Part 4)	0.665	0.715	0.660	**0.670**	0.330
Model 5 (Parts 1, 2, 3, 4 : Part 5)	0.600	0.681	0.680	0.520	0.203
Ensemble Model	0.695	0.759	0.840	0.550	**0.408**

The highest value for each metric is in bold

**Table 4:** Independent test classifying strong enhancers and weak enhancers under 10 trials

No. of Trials	ACC (%)	AUC (%)	SN (%)	SP(%)	MCC
1	0.650	0.728	0.680	0.620	0.301
2	0.710	0.795	0.880	0.540	0.447
3	0.695	0.751	0.920	0.470	0.437
4	0.670	0.749	0.750	0.590	0.344
5	0.660	0.724	0.720	0.600	0.322
6	0.690	0.779	0.810	0.570	0.391
7	0.670	0.736	0.740	0.600	0.343
8	0.660	0.728	0.750	0.570	0.325
9	0.695	0.759	0.840	0.550	0.408
10	0.675	0.735	0.820	0.530	0.366
Mean	0.678	0.748	0.791	0.564	0.368
SD	0.019	0.024	0.076	0.044	0.050

#### Layer 1: enhancer identification

**Fig. 2:**
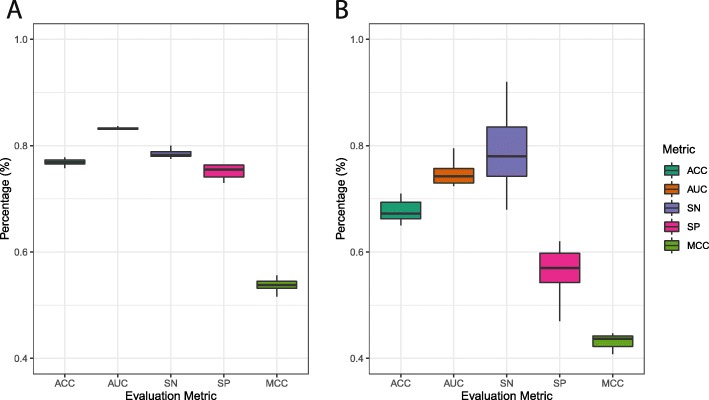
Variation in evaluation metrics from 10 trials of independent test for **a** Layer 1: Enhancer Identication and **b** Layer 2: Enhancer Classication

From five parts split from the development set, after 5 rotations, 5 trained CNN models were obtained to build up an ensemble model. As seen from Table 1, the model accuracy of these models varies between 0.740 and 0.776 with a very small standard deviation. For the AUC, all values are over 0.800 with the highest AUC value of 0.831. Model 3 ends with an opposing result between sensitivity and specificity together with the MCC. Model 3 obtains the highest sensitivity but lowest specificity and MCC compared to others which leads to higher standard deviations in these metrics. In terms of the specificity and MCC, models 1 and 4 were at the first place, respectively. Although some metrics in single CNN models are slightly higher than those of the ensemble model, the ensemble model remains the one having higher efficiency in total examination. In comparison, the specificity of the ensemble model only smaller than that of model 1 while its sensitivity and MCC are only smaller than sensitivity and MCC of models 3 and 4, respectively. To observe the variation in all the evaluation metrics of the ensemble model, 10 trials were done on the independent test set (Fig. 2a and Table 2). The results indicate a very small variation in evaluation metrics among 10 trials with no outlier found, especially the AUC – the least varied metric. The sensitivity is the second lowest metric, followed by the accuracy and specificity. Moreover, the small variation of the MCC implies highly stable prediction over many trials.

#### Layer 2: enhancer classification

Similarly, layer 2 also had its development set split into five parts containing strong enhancers and weak enhancers in an equal ratio in which 4 parts were used as a training set and 1 part was used as a validation set. The ensemble model was finally built up from the five separate CNN models (Table 3). Generally, the variation in evaluation metrics among the 5 models for enhancer classification is greater than those of the five models for enhancer identification. This fact can be explained by the different numbers of samples between the two prediction layers. The sample size of the development set used in layer 1 is obviously significantly larger than the sample size of the development set used in layer 2. Furthermore, differences between enhancers and non-enhancers are more specific than those between strong enhancers and weak enhancers (Fig. 1a). Regardless of their strength, strong enhancers and weak enhancer are still functional enhancers sharing more structural similarities (Fig. 1b). The sensitivity of the ensemble model holds the first place, followed by the AUC, accuracy, and specificity. The MCC of the ensemble model is only over 0.408 but it is the highest value compared to those of 5 single CNN models. Among these evaluation metrics, the AUC is the most stable with the smallest variation compared to the others. The accuracy and AUC of model 1 is higher than those of the rest of the models. Models 3 and 4 have the highest sensitivity and highest specificity, respectively. Although the specificity of the ensemble model is relatively lower than some single CNN models, its high sensitivity promises an effective computational framework because correctly detecting strong enhancers is somehow more important than correctly finding weak ones. The MCC of the enhancer classification model varies more broadly compared to that of the enhancer identification model. To observe the variation in all evaluation metrics of the ensemble model, 10 trials were done on the independent test set to collect data (Fig. 2b and Table 4). The results indicate a quite large variation in sensitivity and MCC among 10 trials. Despite large variation, no outlier is found in all evaluation metrics. The averaged sensitivity of the model is significantly greater than the others but its variation is also higher than the rest of metrics. The MCC is the least varied metric, followed by the AUC, accuracy, and specificity.

### Comparative analysis

Table 5 gives a detailed comparative analysis on the model performance between iEnhancer-ECNN and other existing state-of-the-art methods in previous studies. Except for specificity, iEnhancer-ECNN achieves a significant improvement in model performance based on the rest of the evaluation metrics. For both layers 1 and 2, the proposed method attains slightly lower value compared to other methods introduced in previous studies. On the other hand, remarkable improvements in the AUC, sensitivity, and MCC are observed, especially those in the model of layer 2 with a boost of about 11.0%, 46.5%, and 65.0%, respectively. A significant increase in the MCC indicates that the proposed method considerably improves the model stability as well as overall performance in comparison with the state-of-the-art methods that have relatively small MCCs. This improvement is essential in the model development to confirm the reliability in the binary classification problem. The MCC is considered to be more informative than the accuracy when it considers the proportion of all the four categories (TF, TN, FP, and FN) of the confusion matrix to show a balanced evaluation in model assessment [30]. Undoubtedly, iEnhancer-ECNN performs better than other previously proposed methods with the surge in most of the evaluation metrics.

**Table 5:** Comparative analysis between results of the proposed method and other studies

	Method	ACC	AUC	SN	SP	MCC	Source
Enhancer Identification	iEnhancer-2L	0.730	0.806	0.710	0.750	0.460	Liu et al., 2016
	EnhancerPred	0.740	0.801	0.735	0.745	0.480	Jia and He, 2016
	iEnhancer-EL	0.748	0.817	0.710	**0.785**	0.496	Liu et al., 2018
	iEnhancer-ECNN	**0.769**	**0.832**	**0.785**	0.752	**0.537**	This study
Enhancer Classification	iEnhancer-2L	0.605	0.668	0.470	**0.740**	0.218	Liu et al., 2016
	EnhancerPred	0.550	0.579	0.450	0.650	0.102	Jia and He, 2016
	iEnhancer-EL	0.610	0.680	0.540	0.680	0.222	Liu et al., 2018
	iEnhancer-ECNN	**0.678**	**0.748**	**0.791**	0.564	**0.368**	This study

Values which are significantly higher than the others are in bold

CNNs and OHE have been used in prediction of enhancer-promoter interactions [31] and enhancer identification (layer 1 only) [32]. However, CNNs only can detect local features from OHE. Our method goes beyond that by including global features of the whole sequence through the statistics of 4 different types of *k*-mers. In addition, in ensemble learning, the training sub-sets of all the individual CNN models cover the whole development set. This leads to better generalization of the ensemble model compared to each individual CNN model. This is the reason why iEnhancer-ECNN outperforms other previously proposed methods using the same dataset with significant improvements in most of the evaluation metrics.

## Conclusion

iEnhancer-ECNN using ensembles of convolutional neural networks combining with one-hot encoding and *k*-mers descriptor as the sequence encoding scheme is an efficient computational framework to identify enhancers and classify their strength. The results confirm that the proposed method can robustly and effectively address difficulties in enhancer identification and classification with significant improvements in most of the evaluation metrics compared to other state-of-the-art methods using the same benchmark dataset. In the future, other sequence encoding schemes and advanced ensemble learning methods will be explored to have a trained model to automatically aggregate the predictions of all the CNN models.

## Methods

### Benchmark dataset

**Fig. 3:**
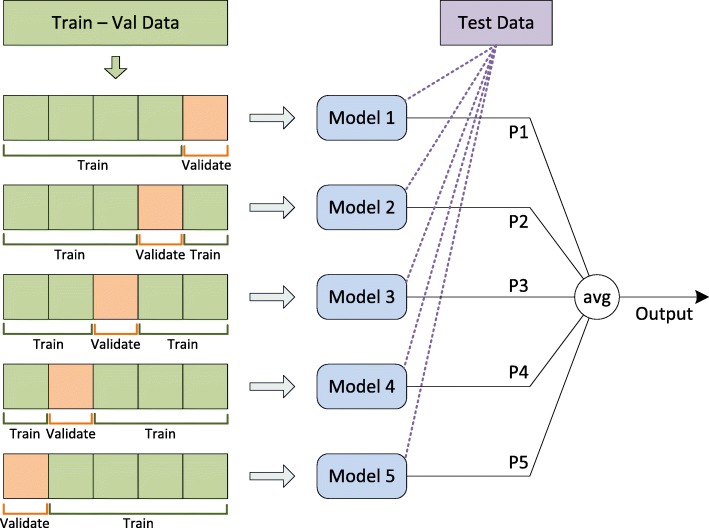
Overview of the model development

The dataset used in our experiments was collected from Liu et al.’s studies [25, 27]. This dataset was also used in the development of iEnhancer-2L [25], EnhancerPred [26] and iEnhancer-EL [27]. In this dataset, information about enhancers from 9 different cell lines was collected and DNA sequences were extracted in the form of short fragments with the same length of 200bp. The CD-HIT software [33] was then used to exclude pairwise sequences whose similarities were more than 20%. The dataset comprises of a development (or cross-validation) set and an independent test set. The development set encompasses 1,484 enhancer samples (742 strong enhancer and 742 weak enhancer samples) and 1,484 non-enhancer samples. The independent test set contains 200 enhancers (100 strong enhancers and 100 weak enhancers) and 200 non-enhancers. Similar to other studies, we used the development set to construct two models for two problems: enhancer identification (layer 1) and enhancer classification (layer 2), then used the independent test set to test the models. For each layer, we first randomly divided the development set into 5 folds (or parts) using stratified sampling. Each fold was in turn used as the validation set while the remaining 4 folds were used as the training set for training a CNN model. Then the five trained CNN models were combined to create an ensemble model for the layer. The ensemble model was then used to test on samples from the independent test set (Fig. 3). This whole process, including data partitioning, model training and model testing, was repeated for 10 times to observe the variation in model performance across 10 trials. Tables 6 and 7 present the data distribution in 5 folds used in model training for layers 1 and 2, respectively.

**Table 6:** Data distribution of 5 parts in the development set for identifying enhancers and non-enhancers

Part	Non-enhancers	Enhancers	
		Strong	Weak
1	301	151	142
2	295	153	146
3	295	148	151
4	292	153	149
5	301	137	154
**Total**	**1484**	**742**	**742**

**Table 7:** Data distribution of 5 parts in the development set for classifying strong enhancers and weak enhancers

Part	Number of enhancers	
	Strong	Weak
1	150	147
2	154	143
3	146	151
4	148	149
5	144	152
**Total**	**742**	**742**

### Sequence encoding scheme

We used one-hot encoding (OHE) and *k*-mer descriptor to encode each input sequence for our CNN model. Every enhancer in this study has a length of 200bp built up by four nucleic acids, including Adenine (A), Guanine (G), Cytosine (C), and Thymine (T). Adenine (A) and Guanine (G) are purines while Cytosine (C), and Thymine (T) are pyrimidines. For OHE, each character was transformed into a new matrix built from a set of 4 binary numbers representing four types of nucleic acids. For each matrix corresponding to a certain type of nucleic acids, there are three values assigned as 0 and one value assigned as 1 (Table 8).

**Table 8:** The corresponding code of each nucleic acid in one-hot encoding

Nucleic Acid	Code
‘A’	[ 1 0 0 0 ]
‘C’	[ 0 1 0 0 ]
‘G’	[ 0 0 1 0 ]
‘T’	[ 0 0 0 1 ]

In addition to OHE, we also used *k*-mers which are the occurrence frequencies of *k* neighboring nucleic acids. With respect to the nucleic acid *N*_*i*_ in a DNA sequence *S* with length *L* (*i*=1..*L* and *L*=200 in this study), in addition to the 4 binary values encoding *N*_*i*_ by OHE, the following 4 values *x,y*,*z,t* were formed and added to the encoding of *N*_*i*_:

- 1-mer feature: $x = \frac {{\# N_{i} \, \text {in} \, S}}{L}$, *N*_*i*_∈{*A,C*,*G,T*}
- 2-mer (right) feature:
  $$ y = \left\{ {\begin{array}{cc} {\frac{{\# N_{i,i + 1} \, \text{in} \, S}}{{L - 1}}} & {\text{if} \,\, i < L} \\ 0 & {\text{if}\, \, i = L} \\ \end{array}} \right. $$
  $$ N_{i,i + 1} \in \left\{ {AA,AC,AG,...,TG,TT} \right\} $$
- 2-mer (left) feature:
  $$ z = \left\{ {\begin{array}{cc} {\frac{{\# N_{i-1,i} \, \text{in} \, S}}{{L - 1}}} & {\text{if} \,\, i > 1} \\ 0 & {\text{if} \,\, i = 1} \\ \end{array}} \right. $$
  $$ N_{i-1,i} \in \left\{ {AA,AC,AG,...,TG,TT} \right\} $$
- 3-mer feature:
  $$ t = \left\{ {\begin{array}{cc} {\frac{{\# N_{i,i+1,i+2} \, \text{in} \, S}}{{L - 2}}} & {\text{if} \,\, i < L-1} \\ 0 & {\text{otherwise }} \\ \end{array}} \right. $$
  $$ N_{i,i+1,i+2} \in \left\{ {AAA,AAC,AAG,...,TTG,TTT} \right\} $$

Thus, each enhancer sample with length 200 is encoded by a matrix of size 200×8.

### CNN architecture

**Fig. 4:**
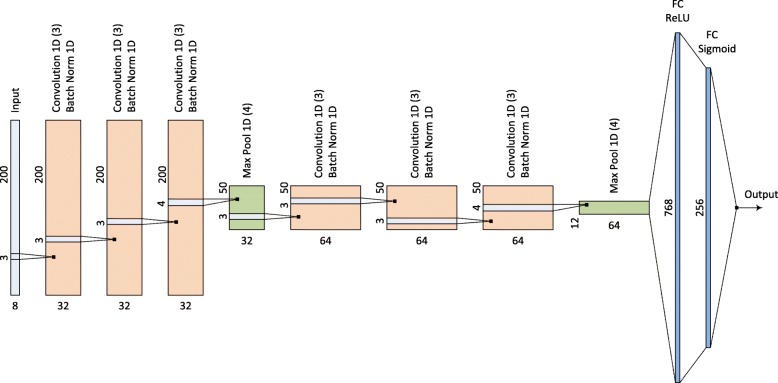
Architecture of the proposed CNN models

Our proposed CNN architecture is described in Fig. 4. The network input is a 200×8 matrix encoding a sequence with length 200. The network consists of six 1-D CNN blocks with batch normalization. Besides, for every three blocks of 1-D CNN, there is one 1-D max pooling layer. After the CNN and the max pooling layers, 768 features are obtained and fed into two fully connected layers with 768 and 256 input neurons using the rectified linear unit (ReLU) and sigmoid activation functions, respectively, to produce a probability of being an enhancer for the input sequence. The same architecture is used to classify strong enhancers and weak enhancers. The models were trained within 20 epochs using the binary cross entropy loss with Adam optimizer [34] and the learning rate of 0.0001. For each CNN model, the optimal network was selected corresponding to the epoch at which the loss on the validation set was minimal.

### Ensemble model

The training process finished with 5 trained CNN models for each layer. For each independent test sample passing through those 5 CNN models, 5 hypotheses (probabilities): *H*_1_, *H*_2_, *H*_3_, *H*_4_, and *H*_5_ were independently computed. We tested the following ensemble methods in order to select the most effective one.

- *The Voting method*: At first, the class of each hypothesis under the threshold of 0.5 were determined to collect 5 class hypotheses. The resultant class was decided based on the frequency of the outcome.
- *The Averaging method*: The hypothesis *H* was calculated as the average value of these five hypotheses under the threshold of 0.5 to give the final result.
- *The Median method*: The hypothesis *H* was calculated as the median value of these five hypotheses under the threshold of 0.5 to suggest the final result.

The threshold of 0.5 was chosen since that value is the default decision threshold in most of classification algorithms. Since our preliminary screening shows the Averaging method worked more effectively compared to others in this study, we adopted this method to construct the ensemble models.

### Model evaluation

To evaluate the model performance, evaluation metrics including accuracy (ACC), sensitivity (SN), specificity (SP), Matthews’s correlation coefficient (MCC), and Area Under the ROC Curve (AUC), were used. TP, FP, TN, and FN are abbreviated terms of True Positive, False Positive, True Negative, and False Negative values, respectively. The mathematical formulas of these metrics are expressed below:

1 $$\begin{array}{@{}rcl@{}} \text{Accuracy}\:(ACC) = \frac{TP+TN}{TP+TN+FP+FN}, \end{array} $$

2 $$\begin{array}{@{}rcl@{}} \text{Specificity}\:(SP) = \frac{TN}{TN+FP}, \end{array} $$

3 $$\begin{array}{@{}rcl@{}} \text{Sensitivity}\:(SN) = \frac{TP}{TP+FN}, \end{array} $$

4 $$\begin{array}{@{}rcl@{}} \textrm{MCC} = \frac{TP{\times}TN-FP{\times}FN}{\sqrt{(TP+FP)(TP\,+\,FN)(TN\,+\,FP)(TN\,+\,FN)}}. \end{array} $$

## Data Availability

The benchmark dataset used in this study were collected from the previous work of Liu et al., 2016. The benchmark dataset were downloaded from the Supplementary Section of the paper entitled “iEnhancer-EL: identifying enhancers and their strength with ensemble learning approach" by Liu et al.. (10.1093/bioinformatics/bty458). Our source code is available at https://github.com/ngphubinh/enhancers.

## Abbreviations

AUC: Area under the ROC curve
CNN: Convolutional neural network
ECNN: Ensemble of CNN
MCC: Matthew’s correlation coefficient
OHE: One-hot encoding
PseKNC: Pseudo k-tuple nucleotide composition
ReLU: Rectified Linear Unit
RF: Random Forest
ROC: Reciever operating characteristic
SVM: Support vector machine
